# Impaired Perceptions and Conspiracy Beliefs about the Way of Emergence of the COVID-19 Infection

**DOI:** 10.3390/ijerph19095557

**Published:** 2022-05-03

**Authors:** Aysen Kutan Fenercioglu, Gunay Can, Nurver Turfaner Sipahioglu, Osman Demir, Semih Gulluoglu, Iyigun Gedik, Gul Ece Altintas, Asena Cosgun, Zekiye Gurcan

**Affiliations:** 1 Department of Family Medicine, Cerrahpasa Medical Faculty, Istanbul University-Cerrahpasa, Istanbul 34098, Turkey; nurverdi@gmail.com (N.T.S.); semih.gulluoglu@istanbul.edu.tr (S.G.); iyigunngedik@hotmail.com (I.G.); 2 Department of Public Health, Cerrahpasa Medical Faculty, Istanbul University-Cerrahpasa, Istanbul 34098, Turkey; gunaycan09@yahoo.fr; 3 Family Healthcare Centre, Kagithane No. 10, Istanbul 34400, Turkey; dr.osman.demir.bdh@gmail.com; 4 Family Healthcare Centre, Fatih No. 7, Istanbul 34104, Turkey; gulecealtintas@gmail.com; 5 Family Healthcare Centre, Kartal No. 7, Istanbul 34862, Turkey; asena.cosgun@hotmail.com; 6 Family Healthcare Centre, Besiktas No. 7, Istanbul 34337, Turkey; z_gurcan@hotmail.com

**Keywords:** COVID-19, coronavirus, perception, conspiracy beliefs

## Abstract

This study aimed to reveal the perceptions and conspiracy theories surrounding the new coronavirus infection. We aimed to explore associations between COVID-19 conspiracy beliefs with recommended health protective attitudes and sociodemographic features among the Turkish population. A questionnaire consisting of seven items about COVID-19 conspiracy theories and perceptions and ten items about attitudes was given to patients and their relatives in five different centres during the second national lockdown in Istanbul. A chi-square test was used to evaluate the associations of disease perceptions and conspiracy beliefs with sociodemographic features and with health protective attitudes. Logistic regression analysis was performed for significant results. Of 483 participants, 242 (50.1%) were found to have a conspiracy belief. Conspiracy theories were more frequent in the participants who were older than 50 years of age (*p* = 0.009) (OR: 1.83) and less frequent in higher education levels (*p* = 0.005) (OR: 0.499). In addition, 6.6% of the participants were infected with COVID-19, but having the infection was found to have no effect on disease perceptions or conspiracy beliefs. Wearing a mask in crowds, avoiding shaking hands and kissing, and washing hands were less frequent among conspiracy believers and participants with impaired perceptions. These results indicate that misconceptions and conspiracy beliefs are driving the adoption of disinformation about the prevention of COVID-19 infection.

## 1. Introduction

Coronaviruses are important human and animal pathogens. Some cases of pneumonia seen originating in the Huanan Seafood Market in Wuhan, China in the last days of 2019 were associated with a new coronavirus epidemic. The new coronavirus, originally named 2019-nCoV, came to be called SARS-CoV-2 in the later stages of the epidemic. The clinical manifestation of this coronavirus disease is named COVID-19: a disease ranging from a mild respiratory illness to a serious condition that can cause considerable mortality [1,2,3,4]. The first case of COVID-19 in Turkey was reported on 11 March 2020, concurrently with the period when COVID-19 was declared a pandemic by the World Health Organization (WHO) [5]. The COVID-19 pandemic has spread to all continents except Antarctica, and 281,808,270 confirmed cases and 5,411,759 deaths have been reported to the WHO from 222 countries and territories as of 27 December 2021 [6]. The actual number of cases is thought to be much higher. It is the largest epidemic after the influenza pandemic of 1918, which affected one-third of the world’s population and killed 50 million people [7].

The spreading dynamics of the disease are an important issue that has been studied. In the early stages of the epidemic, before 1 January 2020, 55% of the patients had a history of exposure to the Huanan Seafood Market; however, after 12 January 2020 this rate decreased to 6% and person-to-person spread was confirmed [1,2,3]. It is thought that the dominant route of spreading is via droplets [8,9,10]. The spread of the infection can also be via an object or surface (fomite) contaminated by an infected person. Thus, the standard recommendations for preventing the spread of infection include regular hand washing, wearing a mask, and social distancing [11,12,13].

Due to the rapid spread of the epidemic all over the world and increasing death rates, the Turkish Ministry of Health took a series of measures, as many countries did, such as curfews, closure of schools and workplaces, and travel restrictions. Shopping centres, restaurants, and entertainment centres were closed. People were forced to stay in their homes. These lockdowns caused a loss of income, increased food prices, and undermined access to non-COVID healthcare services. With the novel coronavirus plaguing the world, there are many uncertainties about the risks of this disease with the possibility of a fatal outcome. The lockdowns and these uncertainties about the nature of the infection have led to a negative impact on individuals, both psychologically and physically, resulting in impaired perceptions and misbeliefs about the disease. One of the main sources of misbelief has been conspiracy theories regarding the origin of the virus itself. Distress, depression, and death anxiety were the first psychological responses to the pandemic and the prevalence of psychological symptoms has greatly increased with time [7,14,15]. The negative perceptions, misconceptions, and misbeliefs seen in these people led to their failure to comply with COVID-related measures. Thus, people with negative perceptions and conspiracy beliefs about the pandemic have also reacted negatively to COVID-19 vaccines and have refused to be vaccinated [16,17]. In their study, Salali et al. revealed that 31% of the Turkish population showed COVID-19 vaccine hesitancy during the time period of early 2020 [18].

Conspiracy theories can be defined as unconfirmed and irrational beliefs involving a malevolent force in the planning of major events where other explanations are more likely [19]. Beliefs about the 9/11 terrorist attacks or the belief that the pharmaceutical industry deliberately spreads disease are examples of conspiracy theories. Such beliefs can have negative health and social effects that have been seen in the past and continue to exist today. A striking example of the impact of conspiracy theories is the HIV epidemic in South Africa, where belief in a conspiracy resulted in government policies with devastating effects on public health. Another example is conspiracy theories about vaccines, which have led to dire consequences such as the re-emergence of infectious diseases including measles, mumps, and rubella [20].

During the coronavirus disease (COVID-19) pandemic, social media platforms have become populated with conspiracy theories; the most popular one has been “COVID-19 was developed in a laboratory” [21]. Even healthcare professionals have believed this theory. In a study carried out in Ecuador, among 252 sampled healthcare workers, 61 (24.2%) believed that the virus was developed intentionally in a laboratory [21]. Evidence suggests that people are more likely to believe conspiracy theories when they feel anxious, powerless, and unable to control outcomes [16,20,21,22]. On the other hand, impaired perceptions about the new coronavirus (COVID-19) are misconceptions regarding the risk of the infection, necessary safety measures, and the consequences of infection [23,24,25,26]. Previous studies indicated a correlation between COVID-19 conspiracies and sociodemographic features such as age, gender, education, and income level [7,16,22]. In a study carried out by Simione et al., belief in conspiracy theories, mistrust in medical information, and mistrust in medicine and science were found to be positively correlated with female sex, age, religious beliefs, psychiatric conditions, and psychological variables, while being negatively correlated with education level [16].

People who believe in conspiracies are less likely to engage in a variety of health behaviours. As examples, people who endorse conspiracy beliefs are less likely to recieve annual physical exams or visit their dentists [27]. Likewise, preliminary evidence suggests that COVID-19 conspiracy beliefs and impaired perceptions about the disease are associated with a lower rate of compliance with health behaviours intended to prevent the spread of COVID-19, such as minimizing time spent outside the home, maintaining social distancing, wearing a mask, and handwashing. In a study conducted in Philadelphia, USA, belief in COVID-19-related conspiracy theories was found to be inversely related to preventive actions, including wearing a mask [28]. In another study from the USA, participants who believed in conspiracies reported complying with public health recommendations to a lesser extent [22]. In the same study, conspiracy beliefs were also found to be associated with reduced COVID-19 public health policy support.

In this study, we aimed to reveal the perceptions and conspiracy theories of the Turkish population about the mechanism of occurrence, the causes, and the consequences of COVID-19 infection and to show their relationship with sociodemographic factors such as age, employment, income, education level, and marital status. We also aimed to reveal the associations of conspiracy beliefs and perceptions with the health protective attitudes of the participants. Thus, we intended to shed light on the main reasons for the Turkish population’s reaction to COVID-19 vaccines and their grounds for disobeying the measures taken by the Turkish government and not following health protective recommendations.

## 2. Materials and Methods

### 2.1. Research Design

This cross-sectional descriptive study was conducted between September and December of 2020, when lockdowns and control measures were augmented due to the increasing spread of COVID-19 in every city in Turkey. A survey consisting of seven questions regarding the perceptions and conspiracy theories about the COVID-19 disease was applied to patients and their relatives in four family healthcare centres and one university centre in Istanbul, the biggest and most populous city in Turkey. The study was carried out in the outpatient clinics of the Department of Family Medicine, Faculty of Medicine, Istanbul University-Cerrahpasa and the Family Healthcare Centres No. 7 Istanbul Kartal, No. 7 Istanbul Besiktas, No. 7 Istanbul Fatih, and No.10 Istanbul Kagithane. The university centre and two of the family healthcare centres were from a low-socioeconomic region whereas the others were from a high-socioeconomic region. Participants consisted of patients and their relatives between the ages of 18 and 65 who were mentally competent, did not have language barriers, and agreed to participate in the study. The participants who did not meet these criteria and those with dementia, psychiatric disorders, or speech and reading disorders were excluded from the study. The nature of the research was explained to the participants before the survey. The survey was applied face to face with the participants and always by the principal investigator of the centre. Each centre had only one principal investigator (A.K.F., O.D., S.G., G.E.A., A.C., and Z.G.). The whole survey took 15 min to complete.

### 2.2. Ethical Permission

Ethics committee approval for the study was obtained from the Clinical Research Ethics Committee of Cerrahpasa Medical Faculty, Istanbul University-Cerrahpasa, (Ref. No. 83045809-604.01.02). The study was also approved by the Scientific Committee of the Turkish Ministry of Health.

All participants signed an informed consent form. The first page of the informed consent form contained information about the purpose of the study, the title of the study, the name of the investigators, and the centres where the study would be conducted. The second page of the informed consent form included information about the responsibilities of the investigator, the rights of participants (e.g., voluntary participation, right to withdraw participation), and the investigator’s contact details.

### 2.3. Description of the Questionnaire

The questionnaire had three sections. The first section included questions about the sociodemographic features/personal factors of the participants, the second section included seven questions on their disease perceptions and beliefs in conspiracy theories about the new coronavirus, and the third section included ten questions about the participants’ health protective attitudes against the new coronavirus.

The sociodemographic features/personal factors measured were: (1) age, gender, and marital status; (2) nationality, place of residence, number of people with whom the participant lives, and mode of transportation; (3) education level, employment, and monthly income; and (4) smoking status, alcohol use, and physical activity. In the questionnaire, the income status of the individuals was classified according to the January 2020 data of the Confederation of Turkish Trade Unions. Accordingly, families with a monthly income below 2219 Turkish Liras were considered to have a low income level, those with a monthly income between 2219 and 7229 Turkish Liras as having a medium income level, and those with a monthly income above 7229 Turkish Liras as having a high income level. The variable age was divided into the following categories: younger than 30 years of age, between 30 and 50 years of age, and older than 50 years of age. In Turkey, general education comprises five years of primary school, including kindergarten, and four years each of secondary school and high school. University education in Turkey is four years in the humanities, social sciences, engineering, and pharmacy, and six years in medicine (Table 1).

In addition to the questions about the participants’ sociodemographic features, the following three questions were also asked the participants to have information about their status of infection with COVID-19: “Have you had COVID-19 infection?”, “Have you had contact with someone who had COVID-19 infection?”, and “How were you diagnosed with COVID-19 infection?”. Three- and four-point Likert scales were used in the scoring of these three questions (Table 2).

The questions about the participants’ perceptions of the disease were as follows: (1) Do you believe that the vaccine for the new coronavirus (COVID-19) will never work and that the human race will be eliminated? (2) Do you believe that the new coronavirus (COVID-19) is no different from an ordinary flu virus and is exaggerated by the media?

The questions regarding conspiracy theories about the emergence of COVID-19 were as follows: (1) Do you believe that the new coronavirus (COVID-19) emerged as a biological weapon (a disease agent that was spread deliberately to kill the masses)? (2) Do you believe that the new coronavirus (COVID-19) was created to produce vaccines and make great profits? (3) Do you believe that the new coronavirus (COVID-19) was produced under laboratory conditions? (4) Do you believe that a secret hand aimed at reducing the world’s rapidly growing population is spreading the new coronavirus (COVID-19)? (5) Do you believe that the new coronavirus (COVID-19) has been produced to revive the mask sales that have decreased considerably in recent years? A three-point Likert scale (yes/no/I have no idea) was used in the scoring of the questions about perceptions and conspiracy theories (Table 3). In addition to these, participants were asked about their fear of COVID-19 disease, and a four-point Likert scale was used in the scoring of this question. With KR-20 (Kuder Richardson) analysis, the internal validity of the conspiracy questionnaire was found to be 0.81.

The third section of the questionnaire included ten questions about health protective attitudes. Those were the questions about using a surgical mask or visor, using hand sanitiser, avoiding shaking hands and kissing, washing hands frequently, social distancing, and cleaning practices. Respondents were asked to respond “yes” or “no” to the attitude questions.

### 2.4. Research Method

The participants who answered “yes” to at least one of the two questions on their perceptions about the COVID-19 infection were considered to have impaired perceptions (Table 3). Likewise, participants who answered “yes” to at least one of the five conspiracy questions were considered to be conspiracy believers based on the methodology of the study by Earnshaw et al. [22]. Then, those with impaired and intact perceptions and those with conspiracy beliefs and disbeliefs were compared in terms of their sociodemographic characteristics and history of COVID-19 infection (Table 4). In Table 5, we compare the associations of belief in conspiracy theories and disease perceptions with the health protective attitudes of the participants.

### 2.5. Statistical Analysis

As the previous studies available for reference were insufficient, sample size estimation for this study was based on our exploration of Epi Info™ StatCalc for the preliminary test. Epi Info™ is a software trademark of the Centres for Disease Control and Prevention (CDC) that is in the public domain and freely available for use. Given a population size of 5 million (the population of Istanbul is 15,462,452 people according to TUIK (Turkish Statistical Institute) [29]), an expected frequency of 50%, an acceptable margin of error of 5%, and five clusters, seventy-seven (77) participants were required to take part in each group; after analyzes of sample sizes, the total required sample size was calculated to be 385. The sample size was increased by 15% to address the possibility that the participants might leave without filling in surveys or be interrupted. Assuming 10% failed to fill the survey correctly (marking the same option in all questions or giving back the survey without markings), the final total sample size was 483. Data were entered and analyzed using SPSS for Windows version 16.0 (SPSS Inc.; Chicago, IL, USA). A chi-square test was used to evaluate the associations between the nonparametric categorical variables such as gender, age, marital status, nationality, education level, monthly income, place of residence, number of people with whom the participant lives, mode of transportation, and the participant’s perceptions/conspiracy theories about COVID-19. Logistic regression analysis was performed for significant results and the exponent of the coefficient was interpreted as the odds ratio (OR) in this statistical evaluation (Table 4). A chi-square test was also used to evaluate the associations of the belief in conspiracy theories and disease perceptions with the health protective attitudes of the participants (Table 5). *p*-values less than 0.05 were considered statistically significant.

## 3. Results

### 3.1. Characteristics of the Study Population

Of 483 participants, 221 (45.74%) were male and 262 (54.24%) were female. Table 1 shows the participants’ sociodemographic characteristics and personal variables. As we aimed to include only the participants who were 18–65 years of age, most of the participants (46.37%) were in the age group 30–50 years. Most of the participants were high school graduates (27.53%) and university graduates (47.82%). Most of them were employed (67.08%), and 15.73% of the employed participants were healthcare workers. Most of the participants were in the middle-income group (54.24%) and lived in an apartment (97.3%) (Table 1). 

### 3.2. Status of Infection with COVID-19

Of the 483 participants, 6.6% had experienced a confirmed COVID-19 infection and 1.9% had experienced a suspected infection without a diagnostic test. Of the participants who had a COVID-19 infection, 90.7% were diagnosed by PCR test, 3.1% by CT-scan, 3.1% by antibody test, and 3.1% by chest x-ray (Table 2). Among 32 participants who had experienced COVID-19 infection, 9.52% reported having come into contact with an infected person.

### 3.3. Perceptions and Conspiracy Beliefs

Most of the participants (56.3%) were very much afraid of being infected by COVID-19, 32.3% were afraid, 7% were slightly afraid, and 4.3% were not afraid. Table 3 shows the participants’ answers to the questions on their perceptions and conspiracy theories about the new coronavirus infection. For the question “Do you believe that the new coronavirus (COVID-19) was produced under laboratory conditions?”, 169 participants (35%) said “yes”, and 144 participants (29.8%) believed that the new coronavirus (COVID-19) emerged as a biological weapon (a disease agent that is spread deliberately to kill the masses). For the question “Do you believe that the new coronavirus (COVID-19) was created to produce vaccines and make great profits?”, 155 participants (32.1%) answered “yes” and 149 participants (30.8%) believed that a secret hand aimed at reducing the world’s rapidly growing population was spreading the new coronavirus (COVID-19). For the question “Do you believe that the new coronavirus (COVID-19) has been produced in order to revive the mask sales that have decreased considerably in recent years?”, 28 participants (5.8%) answered “yes” (Table 3). Considering those who answered “yes” to at least one of these questions, 242 participants (50.1%) were found to have a conspiracy belief about COVID-19 infection and 241 (49.89%) were found to be conspiracy disbelievers.

In perception questions, for the question “Do you believe that the vaccine for the new Coronavirus (COVID-19) will never work and that the human race will be eliminated?”, 33 participants (6.8%) replied “yes” and only 48 participants (9.9%) believed that the new Coronavirus (COVID-19) was not different from an ordinary flu virus and was overly exaggerated by the media for reporting. Considering the “yes” answers to at least one of these perception questions, 67 (13.87%) participants were found to have impaired perception.

### 3.4. Association of COVID-19 Perceptions/Conspiracy Beliefs with Different Variables

Table 4 shows the association between the sociodemographic features/personal variables and COVID-19 perceptions/conspiracy beliefs. In univariate analysis, it was found that conspiracy beliefs were more frequent in the participants who were older than 50 years of age (*p* = 0.009) and in the lower education level group (*p* = 0.005) (Table 4). Logistic regression analysis revealed that the older age had an increasing effect and the higher education level had a decreasing effect on the participants’ conspiracy beliefs about COVID-19. The magnitudes of these effects were as follows: being older than 50 years of age (odds ratio: 1.83, 95% confidence interval: 1.092–3.067) and having an education level higher than secondary school (odds ratio: 0.499, 95% confidence interval: 1.022–2.459) (Table 4). 

**Figure 1:**
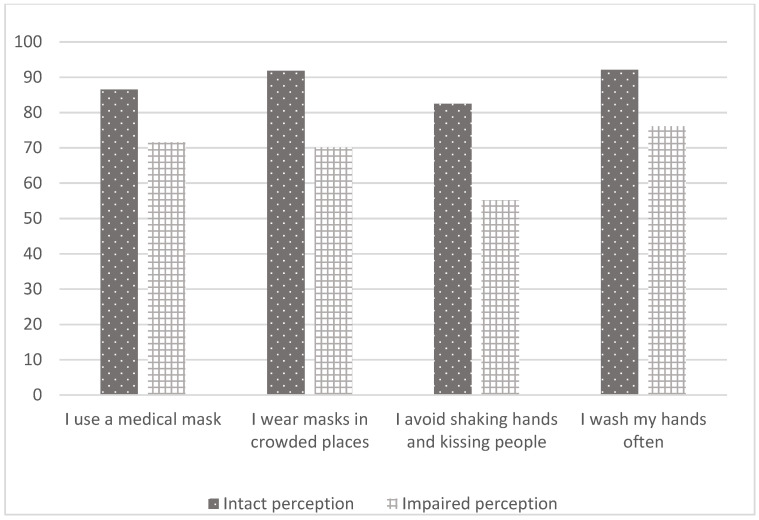
The association of COVID-19 perceptions with attitudes.

**Figure 2:**
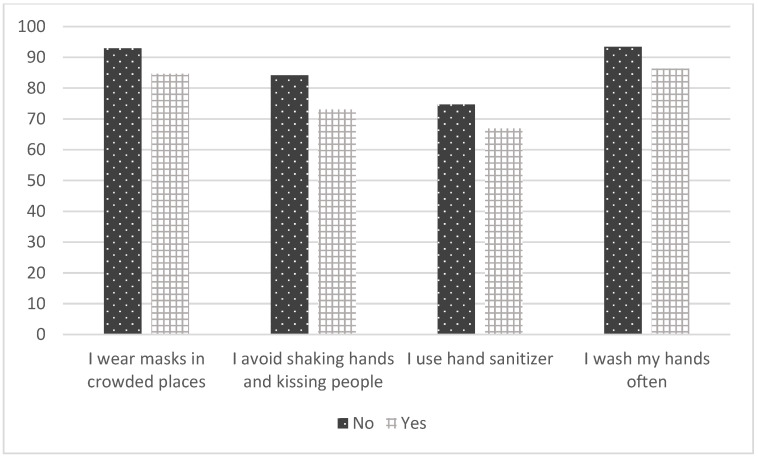
The association of conspiracy belief with attitudes.

Table 5 shows the associations between the conspiracy beliefs/disease perceptions and the health protective attitudes of the participants. It was determined that those with intact perceptions used medical masks more than those with impaired perceptions (*p* = 0.002). In addition, those who did not believe in conspiracy theories were wearing a mask in crowds more than the participants who were conspiracy believers (*p* = 0.004). The rates of those with intact perceptions and those who did not believe in conspiracies who were washing their hands frequently and avoiding kissing people were higher than the other participants. These rates were also found to be statistically significant (Table 5) (Figure 1 and Figure 2). 

## 4. Discussion

The present study revealed that 50.1% of the Turkish population held a conspiracy belief about the new coronavirus (COVID-19) infection. Age and education level were found to be significant in this regard. The results of this study also showed that the rate of the new coronavirus (COVID-19)’ infection was 6.6% between September and December 2020, when there was a second peak of the pandemic in Turkey. Health protective attitudes such as wearing a mask in crowds, avoiding shaking hands and kissing, and washing hands were less frequent among conspiracy believers and participants with impaired perception. This is the first study attempting to measure the perceptions and conspiracy beliefs of the Turkish population about the COVID-19 pandemic.

In a recent study conducted in Jordan with the participation of university students through an online questionnaire, 16.4% of the participants stated that they believed in the role of a conspiracy in the emergence of COVID-19 and 49.9% of them had an inclination to believe this theory. Only one-third of them rejected the idea that COVID-19 was part of a global conspiracy [20]. Correlations of age, education level, and marital status with belief in a conspiracy regarding the origin of the virus did not result in statistically significant differences in this study. Romer et al., from the United States, assessed beliefs in three conspiracy theories in two different time periods with a four-month interval and found that belief in three COVID-19-related conspiracy theories was highly stable across the two periods [28]. This study indicated that individuals younger in age and lower in income and education were more likely to hold conspiracy beliefs about the origins and seriousness of the pandemic. On the other hand, in another study from the United States, conspiracy beliefs were measured with six items and one-third (33%) of participants were found to believe in one or more conspiracies about COVID-19 [22]. The same study revealed that a higher percentage of participants who were younger and who had college degrees believed in conspiracies than participants who disbelieved conspiracies. In our study, we revealed a higher rate of conspiracy beliefs when compared with these previous studies and also showed that conspiracy beliefs were more frequent in the participants who were older than 50 years of age (*p* = 0.009) (OR: 1.83) and less frequent in high school and university graduates (*p* = 0.005) (OR: 0.499). 

In another study carried out in Pakistan, it was reported that, of 1000 participants, 46.0% thought that COVID-19 was a bioweapon, 42.6% believed that it was not, and 11.4% responded that it might be [23]. These results, interestingly, showed that the belief and disbelief in this scenario were equally distributed among participants. Similarly, in our study, 29.8% of the participants believed that the new coronavirus (COVID-19) emerged as a biological weapon, 26.1% believed that it did not, and 44.1% stated that they had no idea about it. Furthermore, 35% of our participants believed that COVID-19 was produced under laboratory conditions, 33.3% believed that it was not, and 31.7% stated that they had no idea about it. Chen et al. conducted a similar survey among 252 sampled healthcare workers in Ecuador and found out that 24.2% of the participants believed that the virus was developed intentionally in a laboratory [21]. This study showed us that healthcare professionals could also believe in conspiracy theories. Our study revealed that 40.8% of health employees in the Turkish population were conspiracy believers.

Bobdey’s article reminds us that the idea of a pandemic triggered by an accidental/intentional release of a bioweapon has existed since the Second World War [24]. The same article also addressed the negative effects of COVID-19 infection on global economies and human psychology. People across the world have been experiencing pandemic fear that worsens their anxiety, leading to mental health disorders. In their study conducted in the Philippines during the early phase of the pandemic, Tee et al. measured participants’ depression, anxiety, and stress levels using the Depression, Anxiety and Stress Scales (DASS-21) and the Impact of Events Scale-Revised (IES-R) [7]. They reported that, of 2037 participants, 16.3% had moderate-to-severe psychological impact, 16.9% had moderate-to-severe depressive symptoms, 28.8% had moderate-to-severe anxiety symptoms, and 13.4% had moderate-to-severe stress signals. The less-educated, single people, children and adolescents, and those who had no children reported high levels of stress, anxiety, depression, and psychological impact. The people in these subgroups are considered to be at greater risk for adverse psychological outcomes during a public health crisis and they may experience a low level of social and emotional support, an increased perceived threat to their wellbeing, and feelings of fear, isolation, and uncertainty [7]. Impaired perceptions about the cause and spread of disease may also increase their levels of anxiety and depression. Our study showed that 52.2% of single participants and 54.3% of participants who were living with more than four people in the same house were conspiracy believers. The increased level of conspiracy beliefs in large families may be related to economic concerns. Kuang et al., from India, studied risk perceptions and changes in behaviours and stress levels during the lockdown. They reported that common fears were related to health and economic concerns, including loss of income (62%), inability to travel freely (46%), and becoming sick (46%) [25]. In our study, 56.3% of participants were found to be very much afraid of being infected by COVID-19, 32.3% were afraid, 7% were slightly afraid, and 4.3% were not afraid.

Vally studied disease perceptions in the United Arab Emirates and found that perceptions about the consequences of the new coronavirus infection and the clarity of public health information were both significantly associated with health protective behaviours [26]. However, the trustworthiness of COVID-19 information in the media and the perceived duration of the pandemic were not found to be significantly associated with health protective behaviours. In our study, 6.8% of the participants believed that the vaccine for the new coronavirus (COVID-19) will never work and the human race will end, and 9.9% believed that the new coronavirus (COVID-19) was not different from an ordinary flu virus and was overly exaggerated by the media for reporting. We found that these impaired perceptions were significantly negatively associated with health protective attitudes such as wearing a mask, washing hands, and avoiding shaking hands and kissing (Table 5).

The study of Earnshaw et al. indicated that participants who believed conspiracies reported complying with public health recommendations to a lesser extent and were less supportive of COVID-19 public health policies than participants who disbelieved conspiracies [22]. Romer et al. also showed that early in the pandemic in the US, COVID-related conspiracy beliefs were inversely related to reporting of both taking preventive actions and intentions to vaccinate against the disease [28]. Similarly, we found significant associations between conspiracy beliefs and preventive attitudes. Mask wearing in public has been increasingly seen as critical to controlling the spread of the coronavirus and those holding conspiracy beliefs were less likely to engage in it. Additionally, avoiding shaking hands and kissing are important elements of social distancing and those who believed in conspiracies were not complying with this recommendation either.

An increasing number of hypothetical scenarios are being produced about the way COVID-19 emerged. This negatively affects the public’s perspective on infection and vaccines. In our study, we found that people over the age of 50 believed in conspiracies more. This explains the low rate of vaccination among people over the age of 65 in Turkey [30]. Nevertheless, we also revealed that the majority of the participants did not have any negative perceptions about COVID-19 vaccines. In the study of Salali et al., 31% of the participants in Turkey were unsure about getting themselves vaccinated for COVID-19 [18], whereas only 6.8% of our participants agreed with the idea that the COVID-19 vaccine will never work. The present study also revealed the importance of education in combating the misbeliefs and misconceptions about COVID-19. Having experienced COVID-19 infection was found to have no effect on the participants’ perceptions or conspiracy beliefs.

### Limitations and Strengths

In our study, we sought to measure how the Turkish people evaluated various scenarios developed to explain the causes of COVID-19 infection at a time when Turkey was experiencing the peak of its infections. To the best of our knowledge, this is the first study to research this particular issue. Our questionnaire was applied face to face in health centres rather than through an online survey. This is another strength. The limitations of our study are that a three-point Likert scale (yes/no/I have no idea) was used in the scoring of the questions about perceptions and conspiracy theories, and a dichotomous scale was used for the attitude questions. Since it was thought that the participants would not want to stay in the health centres for a long time, due to the pandemic, a three-point Likert scale and dichotomous questions that could be filled in a shorter time and would be easier to understand were chosen so that the participants would not give up or make markings that were not reflective of their thoughts. Although four- or five-point Likert scale questionnaires can provide more accurate and detailed information on social and behavioural sciences, there have been similar studies conducted with dichotomous scale questionnaires [20,22]. In addition, our sample of 483 respondents may limit our ability to generalize to the entire Turkish population, but we believe that our study represented all portions of current Turkish society as three of the health centres were from a low-socioeconomic region whereas the others were from a high-socioeconomic region. Most of our participants were high school graduates (27.53%) and university graduates (47.82%) because those who were more educated were more likely to agree to participate in the study. Those who were less educated suggested that they could not understand the questions. This was another weakness of our study. Our conspiracy questions included most of the issues mentioned in previous studies, such as questions about the intentional production of the coronavirus in a laboratory or as a bioweapon, but also included theories that were not seen in any prior study. These questions were about the theories claiming that a secret hand aimed at reducing the world’s rapidly growing population or aimed at reviving mask sales was spreading the new coronavirus. These conspiratorial beliefs tend to attribute power to unseen actors who have interests that diverge from those of the average person. Although some have characterized these conspiracy beliefs as aberrant or reflective of paranoid thinking styles, our findings suggest that they also are common enough to be problematic.

## 5. Conclusions

Misconceptions and conspiracy beliefs are driving the adoption of vaccine avoidance and disinformation about the prevention of COVID-19 infection. News sources should avoid broadcasting and publishing contradictory information that could distort the public’s perceptions. Healthcare institutions should educate the public about the source of COVID-19 infection, its origins, transmission routes, and vaccines. For this purpose, there should be a healthcare authority that can answer the people’s questions in detail at meetings open to the public. Most importantly, studies carried out by healthcare authorities should disseminate clear information about the way that COVID-19 emerged, its transmission routes, and the effects of its vaccines.

## Figures and Tables

**Table 1:** Sociodemographic characteristics and personal variables of the participants.

Sociodemographic Characteristics/Personal Variables (*N* = 483)	Number	%
Gender		
Male	221	45.75
Female	262	54.24
Age		
<30 years of age	142	29.3
30–50 years of age	224	46.37
>50 years of age	117	24.22
Marital Status		
Married	274	56.72
Not married	209	43.27
Education level		
Primary school	63	13.04
Secondary school	56	11.59
Highschool	133	27.53
University	231	47.82
Employment		
Employed	324	67.08
Unemployed	159	32.91
Health employee		
Yes	76	15.73
No	407	84.26
Monthly income		
Low	119	24.63
Medium	262	54.24
High	102	21.11
Nationality		
Turkish	467	96.68
Other nationalities	16	3.31
Place of residence		
Apartment	470	97.3
Other	13	2.69
Number of people she/he lives with		
Single	104	21.53
2 people	110	22.77
3 people	105	21.73
4 and more people	164	33.95
Mode of transportation		
Personal vehicle	210	43.47
Public transport	170	35.19
Pedestrian	58	12
Shuttle	45	9.31
Smoking status		
Non-smoker	317	65.63
<1 pack	120	24.84
≥1 pack	46	9.52
Alcohol use		
Do not use	307	63.56
Social drinker	166	34.36
Use regularly	10	2.07
Physical activity		
Exercise regularly	109	22.56
Exercise seldom	254	52.58
Do not exercise	120	24.84

**Table 2:** Status of infection with COVID-19 of participants (*N* = 483).

Have You Had COVID-19 Infection?	Number	%
Yes	32	6.6
No	381	78.9
I don’t know	61	12.6
I think I had but didn’t have the test done	9	1.9
**Have you had contact with someone who had COVID-19 infection?**		
Yes	46	9.52
No	294	60.86
I don’t know	143	29.60
**How was your COVID-19 infection diagnosed?**		
With PCR test	29	90.7
Antibody test	1	3.1
CT scan	1	3.1
Chest X-ray	1	3.1

**Table 3:** The COVID-19 perceptions and conspiracy beliefs of the participants.

*N* = 483	Yes (%)	No (%)	No Idea (%)
Conspiracy Beliefs			
Do you believe that the new Coronavirus (COVID-19) is produced under laboratory conditions?	169 (35)	161 (33.3)	153 (31.7)
Do you believe that the new Coronavirus (COVID-19) emerged as a biological weapon (a disease agent that spreads deliberately to kill the masses)?	144 (29.8)	126 (26.1)	213 (44.1)
Do you believe the hypotheses that the new Coronavirus (COVID-19) was created to produce vaccines and make great profits?	155 (32.1)	187 (38.7)	141(29.2)
Do you believe that a secret hand aimed at reducing the world’s rapidly growing population is spreading the new Coronavirus (COVID-19)?	149 (30.8)	182 (37.7)	152 (31.5)
Do you believe that the new Coronavirus (COVID-19) has been produced in order to revive the mask sales that have decreased considerably in recent years?	28 (5.8)	389 (80.5)	66 (13.5)
**Perceptions**			
Do you believe the ideas that the vaccine for the new Coronavirus (COVID-19) will never work and that the human race will end?	33 (6.8)	360 (74.5)	90 (18.6)
Do you believe the views that the new Coronavirus (COVID-19) is not different from an ordinary flu virus and is overly exaggerated by the media for reporting?	48 (9.9)	359 (74.3)	76 (15.7)

**Table 4:** The association of COVID-19 perceptions/conspiracy beliefs with personal variables.

Sociodemographic Characteristics/Personal Variables	Conspiracy Beliefs							Perception				
	Conspiracy Disbelief (*n* = 241)		Conspiracy Belief (*n* = 242)		*p* Value ^ǂ^	Odds Ratio (95% CI)	*p* Value *	Intact Perception (*n* = 416)		Impaired Perception (*n* = 67)		*p* Value ^ǂ^
	*n*	%	*n*	%				*n*	%	*n*	%	
**Age**												
<30 years of age	84	59.2	58	40.8		Reference		127	89.4	15	10.6	
30–50 years of age	110	49.1	114	50.9		1.368 (0.884–2.118)	0.159	195	87.1	29	12.9	
>50 years of age	47	40.2	70	59.8	**0.009**	**1.830 (1.092–3.067)**	**0.022**	94	80.3	23	19.7	0.093
**Gender**												
Male	131	50.0	131	50.0				225	85.9	37	14.1	
Female	110	49.8	111	50.2	0.960			191	86.4	30	13.6	0.862
**Marital status**												
Married	110	52.6	99	47.4				183	87.6	26	12.4	
Not married	131	47.8	143	52.2	0.294			233	85.0	41	15.0	0.427
**Education level**												
Secondary school and lower	46	38.7	73	61.3		Reference		100	84.0	19	16.0	
High school and Higher education	195	53.6	169	46.4	**0.005**	**0.499 (1.022–2.459)**	**0.011**	316	86.8	48	13.2	0.446
**Monthly income**												
Low	56	47.1	63	52.9				97	81.5	22	18.5	
Medium	129	49.2	133	50.8				230	87.8	32	12.2	
High	56	54.9	46	45.1	0.484			89	87.3	13	12.7	0.243
**Employment**												
Employed	225	49.9	226	50.1				391	86.7	60	13.3	
Unemployed	84	52.8	75	47.2	0.366			138	86.8	21	13.2	0.767
**Health employee**												
Yes	45	59.2	31	40.8				69	90.8	7	9.2	
No	196	48.2	211	51.8	0.077			347	85.3	60	14.7	0.200
**Place of residence**												
Apartment	234	49.8	236	50.2				405	86.2	65	13.8	
Other	7	53.8	6	46.2	0.773			11	84.6	2	15.4	0.873
**Number of people she/he lives with**												
1	60	57.7	44	42.3				91	87.5	13	12.5	
2–3	58	52.7	52	47.3				93	84.5	17	15.5	
>4	123	45.7	146	54.3	0.093			232	86.2	37	13.8	0.820
**History of past COVID-19**												
Yes	16	50	16	50				25	78.1	7	21.9	
No	225	49.9	226	50.1	0.990			391	86.7	60	13.3	0.175

ǂ Chi-square test. * Multifactorial regression analysis. CI. confidence interval.

**Table 5:** The association of COVID-19 perceptions/conspiracy beliefs with attitudes.

Attitudes	Conspiracy Beliefs					Perception				
	Conspiracy Disbelief (*n* = 241)		Conspiracy Belief (*n* = 242)		*p* Value	Intact Perception (*n* = 416)		Impaired Perception (*n* = 67)		*p* Value
	*n*	%	*n*	%		*n*	%	*n*	%	
**I use a medical mask**										
No	34	14.1	41	16.9		56	13.5	19	28.4	
Yes	207	85.9	201	83.1	0.390	**360**	**86.5**	48	71.6	**0.002** **^ǂ^**
**I use medical gloves**										
No	95	39.4	107	44.2		168	40.4	34	50.7	
Yes	146	60.6	135	55.8	0.285	248	59.6	33	49.3	0.111
**I use safety glasses or a visor**										
No	143	59.3	146	60.3		248	59.6	41	61.2	
Yes	98	40.7	96	39.7	0.824	168	40.4	26	38.8	0.807
**I use an N95 mask**										
No	125	51.9	143	59.1		230	55.3	38	56.7	
Yes	116	48.1	99	40.9	0.11	186	44.7	29	43.3	0.827
**I wear a mask in crowds**										
No	17	7.1	37	15.3		34	8.2	20	29.9	
Yes	**224**	**92.9**	205	84.7	**0.004** **^ǂ^**	**382**	**91.8**	47	70.1	**0.000** **^ǂ^**
**I avoid shaking hands and kissing people**										
No	38	15.8	65	26.9		73	17.5	30	44.8	
Yes	**203**	**84.2**	177	73.1	0.003 ^ǂ^	**343**	**82.5**	37	55.2	**0.000** **^ǂ^**
**I use hand sanitiser**										
No	61	25.3	80	33.1		116	27.9	25	37.3	
Yes	180	74.7	162	66.9	0.061	300	72.1	42	62.7	0.115
**I wash my hands often**										
No	16	6.6	33	13.6		33	7.9	16	23.9	
Yes	**225**	**93.4**	209	86.4	**0.011** **^ǂ^**	**383**	**92.1**	51	76.1	**0.000** **^ǂ^**
**I use detergents for surface cleaning**										
No	200	83.0	194	80.2		338	81.2	56	83.6	
Yes	41	17.0	48	19.8	0.424	78	18.8	11	16.4	0.648
**I use bleach for surface cleaning**										
No	191	79.3	181	74.8		318	76.4	54	80.6	
Yes	50	20.7	61	25.2	0.244	98	23.6	13	19.4	0.453

ǂ Chi-square test.

## Data Availability

The data presented in this study are available on request from the corresponding author.

## Abbreviations

CI	Confidence interval
COVID-19	Coronavirus disease 2019
Epi Info™	is a software trademark of the Centres for Disease Control and Prevention (CDC) that is in the public domain and freely available for use
HIV	Human Immunodeficiency Virus
OR	Odds Ratio
SARS-CoV-2	Severe acute respiratory syndrome coronavirus 2
WHO	World Health Organization
X^2^	Chi-squared test
